# Supplementary material for: Clinical course and potential predictive factors for pneumonia of adult patients with Coronavirus Disease 2019 (COVID-19): A retrospective observational analysis of 193 confirmed cases in Thailand
Source: PLoS Negl Trop Dis. 2020 Oct 16;14(10):e0008806. doi: 10.1371/journal.pntd.0008806 (PMC7592908; doi:10.1371/journal.pntd.0008806)
Supplement: Attachment — Submitted filename: PNTD-D-20-01116 AP .docx [file pntd.0008806.s001.docx]

The authors describes the clinical course and predicting factors of pneumonia in adults diagnosed with COVID-19 receiving care at an infectious disease institute in Thailand. Older age, obesity and fever at presentation were shown to associate with pneumonia. These factors are easily assessed in resource-limited settings. Some comments are as follows:

Major:

1. In the study, initial chest film revealed opacity in 37 patients, but total numbers of pneumonia patients were 75. Pneumonia in this study was defined as the presence of respiratory symptoms and opacity on chest radiography. However, during admission, hospital-acquired pneumonia (HAP) from other pathogens can occur. How the authors excluded HAP from the progression of COVID-19? Also, the frequency of chest radiography to detect lung opacity and/or indication for chest film during hospital admission was not described.
2. ARB use might be collinearity with hypertension in multivariate logistic regression. This might result in no statistical significance of both factors. Some previous studies showed the association of hypertension and COVID-19 pneumonia or having adverse events from the infection.

Minor:

1. The authors use the definition of obesity as BMI ≥30 kg/m^2^. However, obesity is defined as BMI ≥25 kg/m^2^ for Asian in the Asia-Pacific guidelines.
2. Some typos: lines 136 RT-PCT; 264 COVID-10.
