# Supplementary material for: Clinical course and potential predictive factors for pneumonia of adult patients with Coronavirus Disease 2019 (COVID-19): A retrospective observational analysis of 193 confirmed cases in Thailand
Source: PLoS Negl Trop Dis. 2020 Oct 16;14(10):e0008806. doi: 10.1371/journal.pntd.0008806 (PMC7592908; doi:10.1371/journal.pntd.0008806)
Supplement: Attachment — Submitted filename: response to reviewers PLOS NTD.docx [file pntd.0008806.s002.docx]

September 2020

Dr. Johan Van Weyenbergh, Associate Editor

Dr. Victor Santos, Deputy Editor

**PLOS Neglected Tropical Diseases**

Dear Editors and reviewers,

Re: Manuscript reference No. PNTD-D-20-01116

We are very excited to have been given the opportunity to revise our manuscript “Clinical course and potential predicting factors of pneumonia of adult patients with coronavirus disease 2019 (COVID-19): a retrospective observational analysis of 193 confirmed cases in Thailand”. We are pleased to submit the revised version for reconsideration for publication in the **PLOS Neglected Tropical Diseases.**

We highly appreciate the detailed and valuable comments from the reviewers. The suggestions are helpful for us and we shall incorporate most of them in the revised paper. Revisions in the text are shown using yellow highlight for additions, and strikethrough font for deletions. In addition, the manuscript has been entirely reviewed by a native speaker. As below, on behalf of my co-authors, I would like to clarify all the points raised by the reviewers, and we hope the reviewers and the editor will be satisfied with our responses to the comments and the revisions of the original manuscript.

Sincerely yours,

SurasakWiboonchutikul, M.D.

Bamrasnaradura Infectious Diseases Institute,

Department of Disease Control, Ministry of Public Health,

Nonthaburi 11000, THAILAND, E-mail address: surasakwiboon@gmail.com

Tel: +66 (0)2-590-3408, +66 (0)89-918-3909 Fax: +66 (0)2-590-3411

**Response to** **Editor
COMMENT: The manuscript will benefit from a grammatical review by a native speaker.**

**RESPONSE:** We would like to thank you for the recommendation in helping improve our manuscript. The revised manuscript has already been reviewed and edited English by a native speaker.

After a grammatical review by a native speaker, many sentences were revised and a number of awkward words were substituted. However, the original meaning has been kept. The changes for language purpose are highlighted in grey.

In addition, the file of figure 1 has been modified in order to meet PLOS’s requirements of providing a TIFF format for publication.

**Response to Reviewer #1:**

We are thankful to the reviewer for taking the time and energy to help us improve the paper. We appreciate all of your insightful comments. Below are our responses to your comments.

**1. COMMENT: In the study, initial chest film revealed opacity in 37 patients, but total numbers of pneumonia patients were 75. Pneumonia in this study was defined as the presence of respiratory symptoms and opacity on chest radiography. However, during admission, hospital-acquired pneumonia (HAP) from other pathogens can occur. How the authors excluded HAP from the progression of COVID-19? Also, the frequency of chest radiography to detect lung opacity and/or indication for chest film during hospital admission was not described.**

RESPONSE: Thank you for this insightful comment and valuable suggestions. Regarding your first query, 38 laboratory-confirmed COVID-19 patients progressed to have pneumonia after admission. The etiology of pneumonia among these cases was based on the pathogen that was documented in the medical records. Although we considered SARS-CoV-2-infected patients who developed pneumonia were COVID-19 pneumonia, other causes were also ruled out. In the medical records, sputum culture for bacteria, rapid diagnostic test for influenza, and PCR for respiratory virus panel were also performed as a part of diagnostic work up. Only one patient had *Hemophilus parainfluenzae* detected from sputum culture which was considered as co-infection.

For the second query, the median frequency of chest radiograph performed during admission was 3.0 (2.0-5.0) times. Follow-up chest radiograph was done in 155 (80.3%) patients. However, repeated chest radiograph during admission was based on attending physicians’ judgments. Concise answers of your queries have been included in the revised manuscript in yellow highlights.

**Additions to the revised manuscript (revised article with changes highlighted)**

Page 6, line 119-121: Baseline chest radiograph was performed in every patient at admission. The need for follow-up chest radiograph during admission was based on the judgement of each attending physicians.

Page 6, line 137-138: Pneumonia with detection of SARS-CoV-2 RNA from respiratory specimens was considered as COVID 19-associated pneumonia.

Page 15, line 254-256: The median frequency of chest radiograph performed during admission was 3.0 (2.0-5.0). Follow-up chest radiograph was done in 155 (80.3%) of the patients.

Page 15, line 258-261: Among 38 patients who progressed to have pneumonia after admission, diagnostic work up to rule out hospital-acquired pneumonia was performed. Only one patient had *Hemophilus parainfluenzae* detected from sputum culture which was considered as co-infection.

**2. COMMENT: ARB use might be collinearity with hypertension in multivariate logistic regression. This might result in no statistical significance of both factors. Some previous studies showed the association of hypertension and COVID-19 pneumonia or having adverse events from the infection.**

RESPONSE: Thank you for reminding us this important point. We had tested correlation between hypertension and ARB use. We found significant correlation among both factors (r=0.56, p<0.001). We excluded ARB use from multiple logistic regression and performed reanalysis. The significant factors were still the same as previous analysis (increasing age, obesity, and higher temperature at presentation). However, there were a little change in estimated odds ratio, 95% confidence interval, and p-value. The changes in Table 4 and logistic regression analyses of factors associated with COVID-19 pneumonia have been incorporated to the revised manuscript in yellow highlights.

**Additions to the revised manuscript (revised article with changes highlighted)**

Page 9, line 209-211: Collinearity diagnostics were performed for the multivariable logistic regression analysis. A correlation of > 0.5 was considered risk of bias estimation due to collinearity.

Page 22, line 319: Minor changes in estimated odds ratio, 95% confidence interval, and p-value of factors in multiple logistic regression (Table 4).

**3. COMMENT: The authors use the definition of obesity as BMI >30 kg/m2. However, obesity is defined as BMI >25 kg/m2 for Asian in the Asia-Pacific guidelines.**

RESPONSE: We apologized that our definition of obesity was not clear. In this manuscript, obesity was classified according to the World Health Organization (WHO) classification for overweight and obesity (<https://www.who.int/news-room/fact-sheets/detail/obesity-and-overweight>). We have added definition of obesity and inserted a reference in the revised manuscript to make it clearer.

**Addition to the revised manuscript (revised article with changes highlighted)**

Page 6-7, line 140-142: Obesity was classified as body mass index (BMI) ≥ 30 kg/m^2^ according to World Health Organization (WHO) classification for overweight and obesity.

Page 7, line 142 and page 30, line 482-483: Insert reference [21] World Health Organization. Obesity and overweight. [cited 2020 August 28]. Available from <https://www.who.int/news-room/fact-sheets/detail/obesity-and-overweight>.

**Response to Reviewer #2:**

We would like to thank the reviewer for carefully and thoroughly reading our manuscript and for the thoughtful comments that help us to improve the quality of this manuscript. Our response follows.

**1. COMMENT: There were numerous typos in the manuscript, and the writing can and should be substantially improved.**

**2. COMMENT: I am overall positive about this work. Despite its limitations, I think that it provides useful information about COVID-19 patients with mild illness, for whom the authors argue that details are lacking. The writing needs to be significantly improved. There were numerous errors, and many parts of the paper are worded awkwardly. I have included some comments and suggestions, but the authors should not take them as an exhaustive list. I recommend going over the entire paper again carefully to improve the writing.**

**- Page 3, line 50: change "was" to "were"

- Page 3, line 52: change "would" to "can"

- Page 3, line 53: change "and early identify vulnerable patients" to "and identify vulnerable patients in a timely manner"

- Page 3, line 58: change "momentously impact on the health systems of affected countries" to "has had a great impact on the health systems of affected countries"

- Page 3, line 65: change "due to a number" to "due to the fact that a number"

- Page 4, line 77: change "not only more" to "not only the more"

- Page 4, line 78: change "and early identify vulnerable patients" to "and identify vulnerable patients in a timely manner"

- Page 4, line 84: change "was aimed" to "aimed"

- Page 5, line 93: change "were all admitted" to "were admitted"

- Page 5, line 102: change "who" to "who were"

- Page 6, lines 130-131: fix typo

- Page 7, lines 153-154: change "as recovery" to "recoveries"

- Page 7, line 155: change "as death" to "deaths"

- Page 8, line 162: change "defined as patients" to "defined as when patients"

- Page 8, line 174: should the "and" be "or"?

- Page 8, line 177: Don't start a sentence with "p < 0.05". Find a way to reword this.

- Page 8, lines 183-184: reword "were reached the study outcomes"

- Page 8, lines 190: change "which found" to "which was found"

- Page 16, line 246: I would reword this as "Clinical Outcome and Factories Associated with Pneumonia." Using "predicting" can be problematic.

- Page 23, lines 316-317: change "were recovered and able to discharge" to "recovered and were able to discharge"

- Page 23, line 325: "present with mild illness" is awkward wording

- Page 24, line 328: change "single center may not" to "single center and may not"

- Page 24, line 339: change "In conclusions" to "In conclusion"**

RESPONSE: We are delighted and thankful to hear that you think our work is helpful. We accepted our limitation in English writing. We have to apologize for a number of the language errors. Following the reviewer’s recommendation, the revised version of the paper was entirely reviewed by a native speaker and edited before re-submission. A grammatical correction according to your suggestions is highlighted in yellow. The other changes for language purpose reviewed by a native speaker are highlighted in grey.
